# Overcoming the affordability barrier for effective and high quality life saving malaria medicines in the private sector in rural Uganda: the Consortium for ACT Private Sector Subsidy (CAPSS) pilot study

**DOI:** 10.1186/1475-2875-11-S1-O16

**Published:** 2012-10-15

**Authors:** Ambrose O Talisuna, Penny Grevval, Andrew Balyeku, Timothy Egan, Godfrey Bwire, Bram Piot, Renia Coghlan, Maud Lugand, John Bosco Rwakimari, Richard Ndyomugyenyi, Fred Kato, Maria Byangire, Paul Kagwa, Fred Sebisubi, David Nahamya, Angela Bonabana, Susan Mpanga-Mukasa, Peter Buyungo, Julius Lukwago, Allan Batte, Grace Nakanwagi, James Tibenderana, Kinny Nayer, Kishore Reddy, Nilesh Dokvval, Sylvester Rugumambaju, Saul Kidde, Jaya Banerji, George Jagoe

**Affiliations:** 1 Medicines for Malaria Venture-MMV; 2 Malaria Public Health &Epidemiology Cluster, University of Oxford-KEMRI-Wellcome Trust Research Programme and Worldwide Antimalarial Resistance Network-WWARN; 3 Uganda Ministry of Health-Ug-MoH; 4 Uganda National Drug Authority-NDA; 5 Programme for Accessible Health Communication and Education-PACE; 6 Malaria Consortium-MC; 7 Surgipharm Pharmaceuticals; 8 l+Solutions; 9 Management Sciences for Health-MSH

## Background

Artemisinin-based combination therapies (ACTs), the treatment of choice for non-complicated falciparum malaria, are unaffordable and inaccessible in the private sector, yet the private sector is the first port of call for malaria treatment across most of rural Africa. Between August 2007 and May 2010, the Uganda Ministry of Health and the Medicines for Malaria Venture conducted the Consortium for ACT Private Sector Subsidy (CAPSS) pilot study to test whether access to effective malaria treatment could be improved through the provision of highly subsidized ACTs in the private sector.

## Methods

Four intervention districts (Pallisa, Budaka, Kamuli and Kaliro) were purposefully selected to receive branded subsidized medicines-”ACT with a leaf”, while the fifth district (Soroti) acted as the control. Baseline and evaluation outlet exit surveys and retail audits were conducted at all licensed private drug outlets in the intervention and control districts. A survey-adjusted, multivariate logistic regression model was used to analyse the intervention’s impact on: ACT uptake; access to ACTs within 24 hours of symptom-onset; and displacement of sub-optimal antimalarials.

## Results

At baseline, the market share of ACTs was < 1%. However, at evaluation, “ACT with a leaf” had a market share of 69% in the interventions districts. Access to ACTs within 24 hours of symptom onset rose from 0.8% at baseline to 26.2% (95% CI: 23.2 - 29.2%) at evaluation in the intervention districts. In the control district it modestly rose from 1.8% to 5.6% (95% CI: 4.0 - 7.3%). The odds of accessing ACTs within 24 hours in the intervention compared to the control districts was 0.46 (95% CI: 0.08 - 2.68, p = 0.4), at baseline and significantly increased to 6.11 (95% CI: 4.32 - 8.62, p < 0.0001) at evaluation. Children less than 5 years-old had “ACT with a leaf” purchased for them more often than those aged above 5 years. There was no evidence of price gouging.

## Conclusions

Our data demonstrate that a supply-side antimalarial subsidy coupled with an intensive communications campaign significantly increased the uptake of ACTs in the private sector in Uganda.

